# Radial extracorporeal shock wave therapy improves pain and plantar pressure in patients with early-to-mid-stage unilateral knee osteoarthritis: a retrospective before-after controlled study

**DOI:** 10.3389/fmed.2025.1707739

**Published:** 2025-12-15

**Authors:** Tianxiang Yang, Tao Ma, Xueqi Liu, Yi Wang, Yinbin Wang, Xi Chen, Haicheng Wei, DeSheng Chen

**Affiliations:** 1 Third Clinical Medical College of Ningxia Medical University, People's Hospital of Ningxia Hui Autonomous Region, Yinchuan, China; 2 Department of Orthopedics, People's Hospital of Ningxia Hui Autonomous Region, Ningxia Medical University, Yinchuan, China; 3 School of Electrical Information Engineering, Northern Nationalities University, Yinchuan, China

**Keywords:** knee osteoarthritis, extracorporeal shockwave therapy, pain, plantar pressure, gait

## Abstract

**Objective:**

To investigate the effects of radial extracorporeal shockwave therapy (rESWT) on pain and plantar pressure in patients with early-to-mid-stage unilateral knee osteoarthritis (KOA).

**Methods:**

Fifty patients with early-to-mid-stage KOA who received treatment at the Extracorporeal Shockwave Therapy Clinic of the People's Hospital of Ningxia Hui Autonomous Region between January 2025 and June 2025 were selected. General patient data, Visual Analog Scale (VAS) scores for pain, Lequesne index scores, Western Ontario and McMaster Universities Osteoarthritis Index (WOMAC) scores, and plantar pressure results before and after treatment were collected.

**Results:**

VAS scores at all post-treatment assessment time points were significantly decreased compared to pre-treatment values (all *P* < 0.05). Post-treatment, the Heel Force Weight percentage (HFW) decreased, while the High-Pressure Point (HPP), Average Pressure (AP), Foot Body Weight percentage (FBW), Contact Area (CA), and Forefoot Force Weight percentage (FFW) increased compared to pre-treatment values (all *P* < 0.05). Dynamic plantar pressure in the third and fourth metatarsals (M34), fifth metatarsal (M5), arch (FM), and heel (FH) significantly increased after treatment (all *P* < 0.05). The Lysholm score increased and the WOMAC score decreased significantly after treatment (all *P* < 0.05).

**Conclusion:**

rESWT can significantly alleviate knee joint pain and promote the recovery of knee joint function in patients with KOA, thereby reducing the degree of abnormality in plantar pressure distribution.

## 1 Background

Knee osteoarthritis (KOA) is a common chronic degenerative disease with a high incidence rate, affecting approximately 40% of individuals aged 55–64, with over 100 million patients in China (1–4). The primary pathogenesis of KOA involves the degeneration of articular cartilage. It predominantly affects middle-aged and elderly populations, with main clinical symptoms including knee pain accompanied by limited mobility, significantly impacting patients' quality of life. Current primary treatment modalities include conservative management and surgical intervention (5–7). Conservative treatments often yield suboptimal results with recurrent symptoms. Surgical treatments are invasive, costly, and involve a rehabilitation period during which patients may lose work and daily living capacity, requiring considerable nursing care (8). Extracorporeal shockwave therapy (ESWT) is increasingly used in the treatment of musculoskeletal disorders. It is a minimally invasive therapy with small trauma, allowing immediate ambulation after treatment, and is hailed as a “bloodless scalpel” (1, 9). Radial ESWT (rESWT) delivers energy that diminishes from superficial to deep tissues, making it relatively safe (10). Gait is fundamental to human daily activities, and plantar pressure, as a crucial parameter in quantitative gait analysis, can reflect posture, motion status, and gait characteristics under various disease conditions (11, 12). Currently, clinical research on ESWT primarily focuses on its effects on pain, with limited studies investigating its correlation with plantar pressure changes. Therefore, this study aims to: (1) evaluate the impact of rESWT on plantar pressure in patients with early-to-mid-stage KOA; and (2) provide a theoretical basis for understanding the dual role of ESWT technology in pain management and biomechanical recovery.

## 2 Methods

### 2.1 Patients and study design

Fifty patients with KOA (Kellgren-Lawrence grade II–III) who were treated at the Extracorporeal Shockwave Therapy Clinic of the People's Hospital of Ningxia Hui Autonomous Region between January 2025 and June 2025 were selected based on weight-bearing anteroposterior and lateral knee radiographs. The inclusion criteria were: (1) those who met the “Chinese Guidelines for the Diagnosis and Treatment of Osteoarthritis (2021 Edition)” (13) knee osteoarthritis diagnostic criteria; (2) no history of surgery or trauma in either lower limb; (3) duration of pain before treatment exceeding 1 month and no intake of any analgesic medications within the week prior to treatment; (4) absence of foot diseases or developmental abnormalities; and (5) patients and their families provided informed consent for participation in this study and signed an informed consent form. The exclusion criteria included: (1) patients with congenital developmental deformities of the lower limbs or hip joint; (2) patients with bilateral lesions or severe knee joint deformities (14); (3) patients with fibromyalgia; (4) patients with severe internal medical diseases; and (5) patients unable to complete the treatment course or with incomplete follow-up data. This study was approved by the Ethics Committee of Ningxia Hui Autonomous Region People's Hospital (approval number: [2024]-KJCG-001).

### 2.2 Treatment method

A radial extracorporeal shockwave device (Manufacturer: Uniphy Elektromedizin GmbH & Co. KG, Model: 300) was used. Treatment sites were first identified, primarily located in areas that elicited pain during knee flexion/extension, ligament stretching, or upon application of pressure. Key treatment areas included the medial and lateral knee dimples (joint line), the medial and lateral femoral condyles, and the suprapatellar pouch (6). The selected areas were marked prior to treatment. Ultrasound coupling gel was applied to the treatment area. The shockwave parameters were set at a frequency of 6–8 Hz and an intensity ranging from 2.0 to 3.0 bar. The applicator head of the radial shockwave device was aligned with the marked treatment points, carefully avoiding major blood vessels and nerves. Treatment commenced at a lower energy level and was gradually increased to a intensity tolerable for the patient. Each treatment point received 2,000–4,000 shocks. Sessions were conducted once per week for four consecutive weeks (2, 15).

### 2.3 Data acquisition

The study utilized an eMat plantar pressure distribution testing system. The system primarily consists of a pressure plate, an Fmap Analysis System upper-computer software, and a walking indication runway, enabling real-time display and storage of acquired pressure data. This system was designed by the team of Professor Wei Haicheng at the School of Electrical and Information Engineering, North Minzu University (16).

#### 2.3.1 Static plantar pressure measurement

During static measurement, subjects were instructed to rest adequately before the experiment to ensure stable physical condition. Subsequently, subjects stood barefoot on the effective measurement area of the pressure plate, with feet naturally apart, arms hanging naturally, maintaining body stability.

#### 2.3.2 Dynamic plantar pressure measurement

For dynamic plantar pressure measurement, subjects removed their shoes and socks and walked naturally toward the pressure plate from a distance of 2 m, returning from the opposite side. Throughout the process, subjects were instructed to maintain a natural gait with arms swinging naturally. The instrument automatically captured and recorded dynamic plantar pressure distribution data. Subjects were required to repeat the measurement process at least three times to obtain more precise results.

### 2.4 Outcome measures

Research data were collected and recorded by two physicians blinded to the intervention.

#### 2.4.1 Analgesic effect

Visual Analog Scale (VAS) scores for pain were assessed before treatment, after the first treatment, 1 week after the first treatment, after the fourth treatment, and 4 weeks after the completion of treatment.

#### 2.4.2 Plantar pressure parameters

(1) Static plantar pressure characteristics: included High-Pressure Point (HPP), Average Pressure (AP), Contact Area (CA), Foot Body Weight percentage (FBW), Heel Force Weight percentage (HFW), and Forefoot Force Weight percentage (FFW). Table 1 details the specific meanings of these static gait characteristic analysis indicators; and (2) Dynamic plantar pressure characteristics: the dynamic plantar pressure distribution was divided into different regions: big toe (T), first metatarsal (M1), second metatarsal (M2), third and fourth metatarsals (M34), fifth metatarsal (M5), arch (FM), and heel (FH). Data were collected before treatment and 4 weeks after the final treatment.

**Table 1:** Static plantar pressure indicators and their definitions.

**Indicator**	**Abbreviation**	**Definition**
High-pressure point	HPP	The force exerted at the maximum pressure point on the sole while standing, calculated as the ratio of the force magnitude to the area of that point, in kg/cm^2^.
Average pressure	AP	The average force exerted on the left (or right) foot during standing, calculated as the total force divided by the total contact area, in kg/cm^2^.
Contact area	CA	The total contact area of the left (or right) foot during standing, in cm^2^.
Foot bearing weight as a percentage of body weight	FBW	The percentage of body weight supported by the left (or right) foot during standing, in %.
Heel force weight as a percentage of total foot pressure	HFW	The percentage of the force exerted by the heel relative to the total force exerted by the entire foot during standing, in %.
Forefoot force weight as a percentage of total foot pressure	FFW	The percentage of the force exerted by the forefoot relative to the total force exerted by the entire foot during standing, in %.

#### 2.4.3 Clinical scoring indicators

Lequesne Index score and Western Ontario and McMaster Universities Osteoarthritis Index (WOMAC) score. Collection times were synchronized with plantar pressure measurements.

### 2.5 Statistical methods

Data analysis was performed using SPSS software (version 24.0). Measurement data were tested for normality using the Shapiro-Wilk test. Normally or approximately normally distributed data are expressed as mean ± standard deviation (*x* ± s). Non-normally distributed data are expressed as median and interquartile range [M (P25, P75)]. Comparisons within groups were performed using paired *t*-tests or non-parametric tests (Wilcoxon signed-rank test). Count data are expressed as ratios (%). A *P*-value < 0.05 was considered statistically significant.

## 3 Results

### 3.1 Patient characteristics

The general characteristics of the patients, including gender, age, and BMI, are shown in Table 2.

**Table 2:** Baseline characteristics of patients.

**Characteristic**	**Value**
**Sex**	
Male	22 (44.00%)
Female	28 (56.00%)
Age (years)	65.36 ± 5.22
BMI (kg/m^2^)	25.42 ± 3.71
**KL grade**	
II	26 (52.00%)
III	26 (52.00%)
Disease duration (months)	12 (3, 12)

Data are presented as x ± s or M (P25, P75) based on the Shapiro-Wilk normality test (α = 0.05).

### 3.2 Analgesic effects

VAS scores at all post-treatment assessment time points showed statistically significant differences compared to the pre-treatment score (all *P* < 0.05; Table 3).

**Table 3:** Comparisons of VAS scores before and after treatment.

**Time point**	**VAS scores**	*Z* ^*^	*P* ^*^
Pre-treatment	5 (5, 6)	–	–
After 1st treatment	3 (3, 4)	−6.038	< 0.001
1 week after 1st treatment	3 (3, 4)	−6.069	< 0.001
After 4th treatment	3 (3, 4)	−6.016	< 0.001
4 weeks post-treatment	3 (3, 3)	−6.172	< 0.001

Data are presented as x ± s or M (P25, P75) based on the Shapiro-Wilk normality test (α = 0.05).

^*^Comparison with preoperative.

### 3.3 Static plantar pressure characteristics

Post-treatment, HFW decreased, while HPP, AP, FBW%, CA, and FFW increased compared to pre-treatment values. All comparisons were statistically significant (all *P* < 0.05; Table 4).

**Table 4:** Comparison of static plantar pressure characteristics before and after treatment.

**Indicator**	**Pre-treatment**	**Post-treatment**	***Z***	***P***
HPP (kg/cm^2^)	1.23 ± 0.26	1.77 (1.56, 2.06)	−6.039	< 0.001
AP (kg/cm^2^)	0.29 (0.28, 0.33)	0.40 (0.30, 0.47)	−3.981	< 0.001
CA (cm^2^)	58.75 (56.63, 77.75)	75.25 (65.75, 86.38)	−3.012	< 0.001
FBW (%)	39.60 (32.75, 50.30)	48.80 (46.80, 51.40)	−4.190	< 0.001
HFW (%)	71.15 (57.50, 77.40)	59.50 (46.85, 75.68)	−2.689	0.007
FFW (%)	23.70 (17.30, 37.50)	36.50 (19.50, 48.15)	−2.775	0.006

Data are presented as x ± s or M (P25, P75) based on the Shapiro-Wilk normality test (α = 0.05).

### 3.4 Dynamic plantar pressure characteristics

Post-treatment, dynamic pressure in the big toe (T), first metatarsal (M1), and second metatarsal (M2) regions showed no statistically significant difference compared to pre-treatment (all *P* > 0.05). Dynamic pressure in the third and fourth metatarsals (M34), fifth metatarsal (M5), arch (FM), and heel (FH) regions significantly increased after treatment (all *P* < 0.05; Table 5).

**Table 5:** Comparison of static plantar pressure characteristics before and after treatment.

**Plantar region**	**Pre-treatment**	**Post-treatment**	***Z***	***P***
T: big toe	73.50 (71.55, 81.15)	81.05 (52.40, 88.70)	−0.130	0.896
M1: first metatarsal	79.80 (77.50, 81.30)	83.00 (72.00, 87.40)	−0.936	0.349
M2: second metatarsal	79.80 (73.40, 84.90)	82.20 (73.50, 85.12)	−0.068	0.946
M34: third and fourth metatarsals	79.51 ± 4.39	83.20 (79.10, 87.00)	−3.350	0.001
M5: fifth metatarsal	79.60 (74.45, 81.38)	81.30 (75.68, 84.70)	−2.409	0.016
FM: arch	70.90 ± 6.85	55.50 (46.50, 69.85)	−4.626	< 0.001
FH: heel	71.95 (65.38, 78.33)	51.30 (43.75, 59.00)	−5.676	< 0.001

Data are presented as x ± s or M (P25, P75) based on the Shapiro-Wilk normality test (α = 0.05).

### 3.5 Clinical scores

The Lysholm score significantly increased, and the WOMAC score significantly decreased after treatment compared to pre-treatment values (all *P* < 0.05; Table 6).

**Table 6:** Comparison of clinical scores before and after treatment.

**Indicator**	**Pre-treatment**	**Post-treatment**	***Z***	***P***
Lysholm score	73.00 (69.75, 78.00)	74.00 (71.00, 79.00)	−2.030	0.042
WOMAC score	113.00 (107.00, 118.00)	108.00 (103.75, 114.25)	−2.577	0.010

## 4 Discussion

With increasing societal focus on the elderly population and the rising proportion of older adults, enhancing their daily quality of life is particularly important. KOA is a prevalent disease among the elderly and is a significant cause of mobility impairment, severely affecting their quality of life (5, 17–19). KOA not only inflicts substantial physical and psychological harm on patients but also often leads to disability, imposing a heavy economic burden on their families and society (3, 20, 21). Current treatment for KOA follows a stepped-care approach. For early-to-mid-stage KOA, pharmacological therapy or traditional Chinese medicine is often used. While pharmacological treatments can relieve pain and improve function in the short term, they require long-term oral administration, and symptoms tend to recur after discontinuation (22). ESWT is a minimally invasive physical therapy that utilizes high-pressure generated high-speed pulsed waves to impact the affected area. It promotes metabolism, analgesia, anti-inflammation, as well as tissue regeneration and repair through mechanical, thermal, and cavitation effects (1, 23). ESWT may promote cartilage regeneration by activating chondrocytes, reduce osteophyte formation, increase osteoblast activity, decrease calcitonin gene-related peptide in subchondral bone, and alleviate chronic inflammatory activity throughout the joint by downregulating inflammatory cytokines, potentially reversing the KOA pathological process to some extent (24). Extracorporeal shockwave therapy may promote cartilage regeneration and differentiation by activating chondrocytes and mitigate chronic joint inflammation by downregulating inflammatory cytokines (25). In our study, VAS scores improved at all time points after rESWT, indicating its significant efficacy in alleviating knee pain. Furthermore, no significant adverse reactions were observed during the course of ESWT treatment. Patients were able to stand and walk immediately after treatment without pain, which is crucial for improving their quality of life.

Plantar pressure characteristics can reflect information regarding postural control balance, different motion states, and gait characteristics under various disease conditions. The main symptoms of KOA include knee pain, stiffness, decreased muscle strength around the joint, and joint deformity. Abnormal loading of the knee joint can affect foot loading (26). During walking, the inability to effectively absorb and dissipate impact forces may transfer more force to other parts of the lower extremity, leading to abnormally increased or decreased pressure in specific regions of the foot, manifesting as abnormal pressure distribution (27). This can result in reduced stride length, increased cadence, altered walking posture, and abnormal gait (28). Gait is fundamental to human daily activities, and plantar pressure serves as a key parameter in quantitative gait analysis, reflecting posture, motion status, and gait characteristics under disease conditions (11, 12). In normal gait, plantar pressure distribution is even, effectively dispersing the weight borne by the foot, avoiding pain and injury caused by excessive focal pressure, and maintaining gait stability (29). When the gait of KOA patients alters, the distribution and magnitude of plantar pressure also change, exhibiting significant gait characteristic differences. Therefore, analyzing plantar pressure characteristics is important for understanding the mechanisms of the gait cycle and for assessing the gait of KOA patients.

During quiet standing, healthy individuals typically bear approximately half of their body weight on each foot (30). However, in KOA patients, pain in the affected knee alters the body's center of gravity, consequently affecting foot loading. In this study, post-treatment, the affected limb's HFW% decreased, while HPP, AP, FBW, CA, and FFW increased compared to pre-treatment. These results suggest that KOA patients might avoid excessive weight-bearing on the affected foot during standing due to pain, leading to a shift in body weight toward the sound side and an abnormally low weight-bearing percentage on the affected foot. rESWT improved patients' pain, increasing the pressure threshold tolerable by the affected foot, elevating the average pressure, enlarging the contact area, increasing the percentage of body weight borne, and creating a more balanced pressure distribution between the rearfoot and forefoot. This helped normalize the body's center of gravity shift previously caused by pain. In dynamic plantar pressure, the increased pressure in the M34 region of the affected limb after treatment suggests that improved pain allows the affected foot to provide stronger propulsion during the push-off phase of gait. Furthermore, the pressure in the M5 region was higher post-treatment, while values in the FM and FH regions were lower post-treatment. The Lysholm and WOMAC scores showed significant improvement. This phenomenon indicates that the medial plantar dynamic pressure on the affected side improved after rESWT, with a distribution shift toward the anterolateral aspect. This change is likely due to the alleviation of knee pain following rESWT, leading to an improvement in the pain-induced “avoidance gait,” allowing for forward shifting of the center of gravity which facilitates walking. This reduces self-protective mechanisms and compensatory clinical manifestations caused by pain, resulting in better clinical scores and plantar pressure parameters that tend toward normality post-treatment.

This study has several limitations. First, multiple statistical comparisons were performed across various outcome measures, which increases the risk of Type I errors (false positives); therefore, the significant findings related to secondary outcomes, particularly the detailed plantar pressure parameters, should be interpreted with caution. Second, while we have demonstrated statistically significant improvements, a comprehensive interpretation regarding the Minimal Clinically Important Difference (MCID) is constrained. For the patient-reported outcomes, although the reduction in VAS pain score appears substantial, the lack of a patient-anchored assessment prevents us from definitively concluding that each individual patient perceived the change as meaningful. For functional scores like the WOMAC and Lysholm indexes, the established MCID values in the literature vary considerably. Therefore, the clinical importance of the observed functional improvements requires cautious interpretation and validation against population-specific MCID benchmarks. Most notably, for the novel biomechanical parameters of plantar pressure, there is a pronounced absence of universally accepted MCID values in individuals with KOA. This makes it challenging to definitively state whether the observed quantitative changes in pressure distribution translate into a subjectively perceptible improvement in gait quality or stability for the patients. Third, the assessment indicators used were not comprehensive and did not include other gait parameters. Fourth, the follow-up period was relatively short. Longer-term observation is needed to verify whether early biomechanical improvements translate into sustained quality-of-life enhancements. Fifth, although patients with severe systemic diseases were excluded, common comorbidities such as mild lumbar spinal stenosis or early-stage diabetes were not systematically assessed or stratified. Such conditions might interfere with plantar pressure recovery by altering movement patterns or pain perception. Future studies should incorporate comorbidity indices and baseline gait assessments to control for these potential confounding factors. Finally, we acknowledge that the single-center, retrospective before-after study design may not fully distinguish the “treatment effect” from other factors like the “time effect” or natural history. However, it is important to emphasize that KOA is a chronic degenerative condition with minimal natural fluctuation over short periods. Our inclusion criteria required pain duration greater than 1 month pre-treatment. The primary outcome measure, plantar pressure, is objective, quantifiable, and less susceptible to subjective bias. Furthermore, this study involved treating all participants, avoiding the ethical issue of withholding treatment from a control group. This study serves as preliminary exploratory research to verify whether rESWT effectively improves plantar pressure in KOA patients. Future large-scale, multicenter randomized controlled trials should combine patient-reported outcome measures with biomechanical gait analysis. This comprehensive approach will more fully reveal the dual impact of interventions on functional performance and patient-perceived quality of life. The minimal clinically important difference (MCID) for kinematic indicators in this population also needs further clarification, which will aid in assessing the degree of benefit patients receive from ESWT. The rESWT protocol and plantar pressure analysis scheme employed in this study are standardized and reproducible, facilitating future validation in more diverse populations and multi-center settings.

## 5 Conclusions

In summary, this study demonstrates that rESWT is significantly effective in treating early-to-mid-stage knee osteoarthritis. It shows notable effects in alleviating pain, improving knee joint function, and normalizing plantar pressure distribution. As a non-invasive physical therapy method, rESWT offers advantages such as minimal trauma, no significant adverse reactions, and immediate ambulation post-treatment, making it worthy of clinical promotion and application.

## Data Availability

The raw data supporting the conclusions of this article will be made available by the authors, without undue reservation.
